# Supplementary material for: Effects of residential mobility and migration on standards of living in Dar es Salaam, Tanzania: A life-course approach
Source: PLoS One. 2020 Sep 29;15(9):e0239735. doi: 10.1371/journal.pone.0239735 (PMC7523954; doi:10.1371/journal.pone.0239735)
Supplement: S1 File — (DOCX) [file pone.0239735.s001.docx]

**S1: Sequence Index and Parallel Coordinate Plots for Migrants and Non-migrants**

***
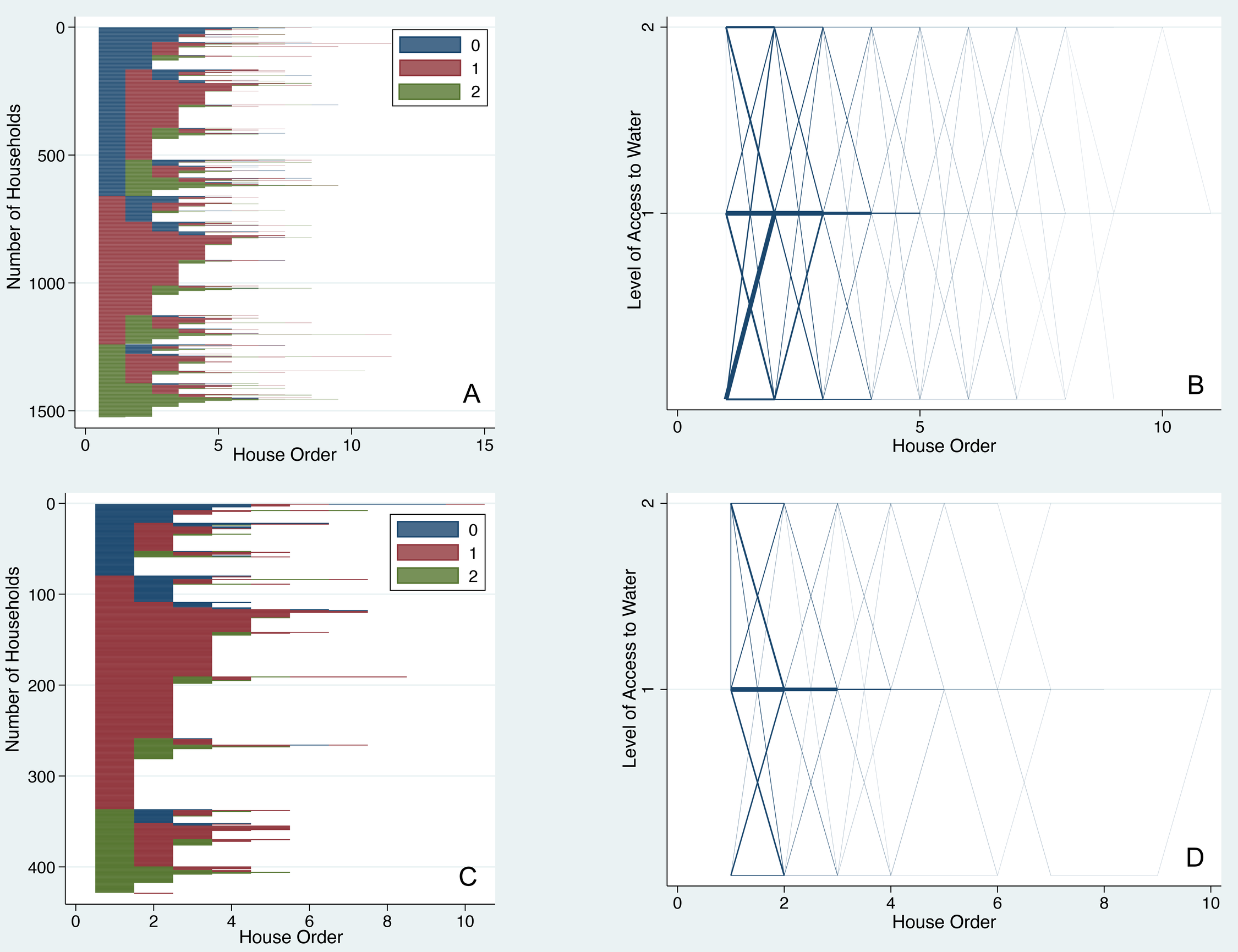
***

**Fig S1.1. Dynamics of access to water for migrants and non-migrants with residential moves**

(A) Sequence index plot for levels of access to water of migrants: individual housing trajectories as horizontal lines grouped by initial conditions. (B) Parallel-coordinate plot for levels of access to water for migrants: line thickness shows volume of flow for a given housing trajectory. (C) Sequence index plot for levels of access to water of non-migrants: individual housing trajectories as horizontal lines grouped by initial conditions. (D) Parallel-coordinate plot for levels of access to water for non-migrants: line thickness shows volume of flow for a given housing trajectory.

**
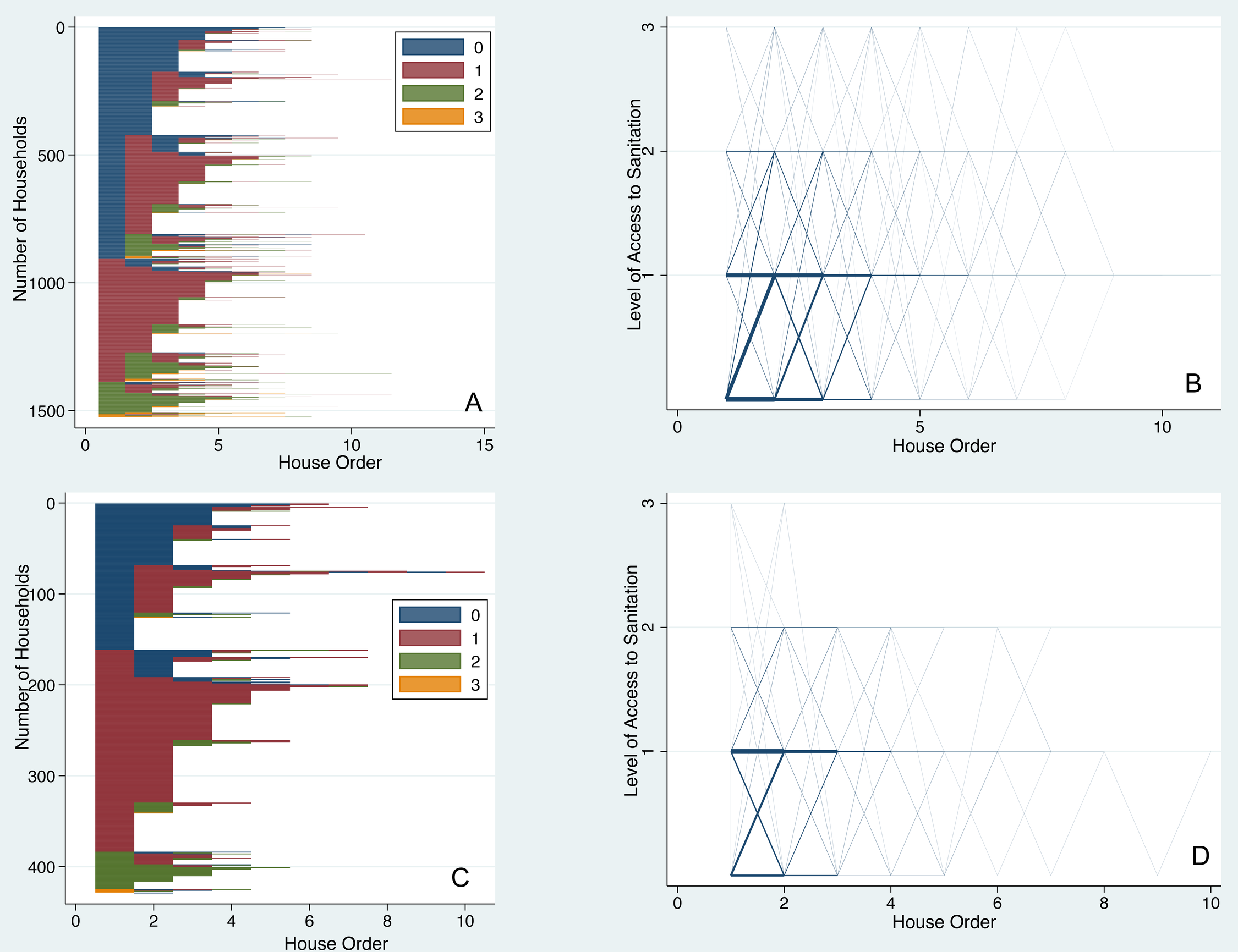
**

**Fig S1.2. Dynamics of access to sanitation for migrants and non-migrants with residential moves**

(A) Sequence index plot for levels of access to sanitation of migrants: individual housing trajectories as horizontal lines grouped by initial conditions. (B) Parallel-coordinate plot for levels of access to sanitation for migrants: line thickness shows volume of flow for a given housing trajectory. (C) Sequence index plot for levels of access to sanitation of non-migrants: individual housing trajectories as horizontal lines grouped by initial conditions. (D) Parallel-coordinate plot for levels of access to sanitation for non-migrants: line thickness shows volume of flow for a given housing trajectory.

**
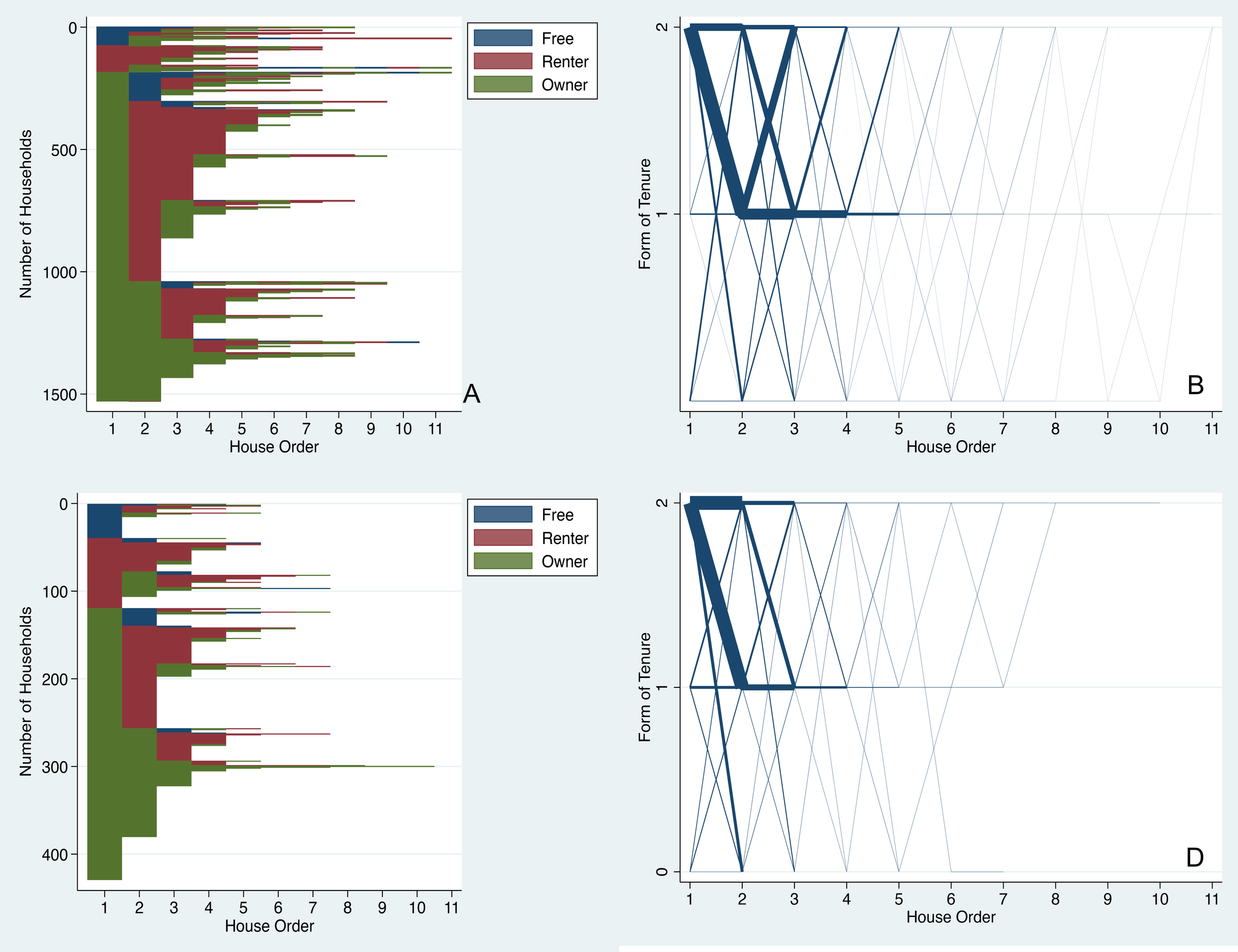
**

**Fig S1.3. Dynamics of housing with durable structure for migrants and non-migrants with residential moves**

(A) Sequence index plot for levels of durable structure of migrants: individual housing trajectories as horizontal lines grouped by initial conditions. (B) Parallel-coordinate plot for levels of durable structure for migrants: line thickness shows volume of flow for a given housing trajectory. (C) Sequence index plot for levels of durable structures of non-migrants: individual housing trajectories as horizontal lines grouped by initial conditions. (D) Parallel-coordinate plot for levels of durable structure for non-migrants: line thickness shows volume of flow for a given housing trajectory.

**
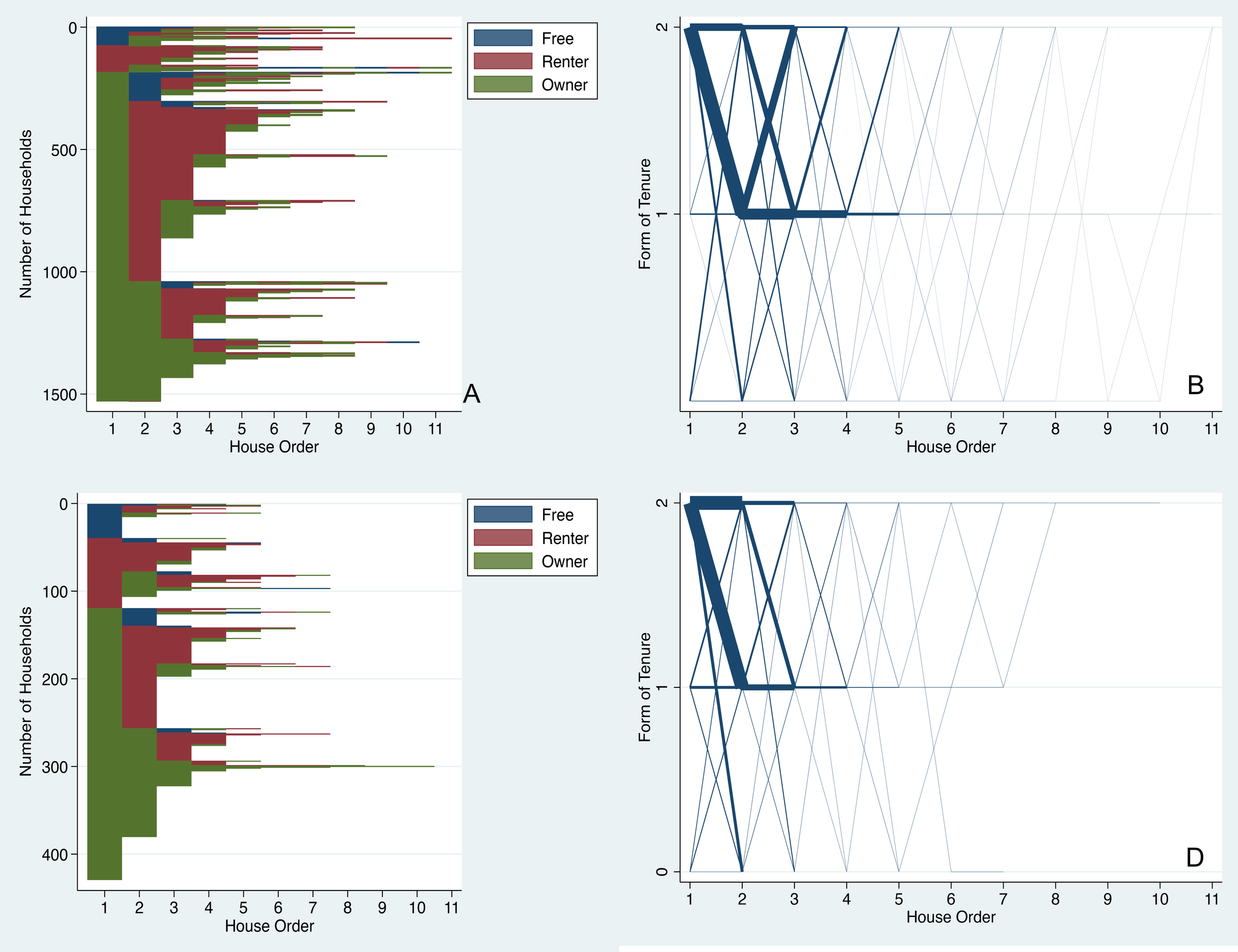
**

**Fig S1.4. Dynamics of tenure forms for migrants and non-migrants with residential moves**

(A) Sequence index plot for forms of tenure for migrants: individual housing trajectories as horizontal lines grouped by initial conditions. (B) Parallel-coordinate plot for forms of tenure for migrants: line thickness shows volume of flow for a given housing trajectory. (C) Sequence index plot for forms of tenure non-migrants: individual housing trajectories as horizontal lines grouped by initial conditions. (D) Parallel-coordinate plot for forms of tenure for non-migrants: line thickness shows volume of flow for a given housing trajectory.
