# Supplementary material for: Effects of residential mobility and migration on standards of living in Dar es Salaam, Tanzania: A life-course approach
Source: PLoS One. 2020 Sep 29;15(9):e0239735. doi: 10.1371/journal.pone.0239735 (PMC7523954; doi:10.1371/journal.pone.0239735)
Supplement: S2 File — (DOCX) [file pone.0239735.s002.docx]

**S2: Sequence Index and Parallel Coordinate Plots for Shanty and Non-shanty Residents**

**
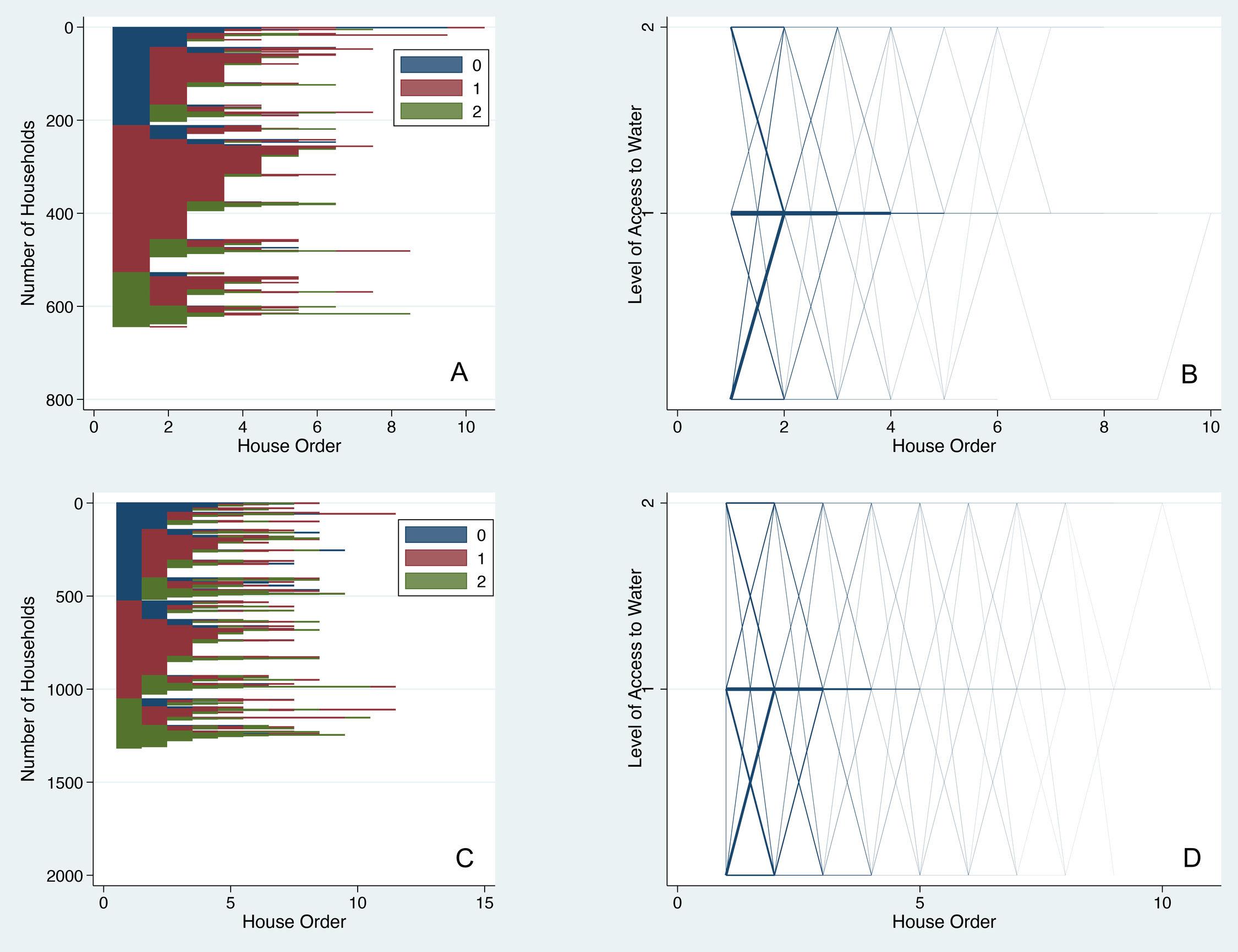
**

**Fig S2.1 Dynamics of access to water for shanty and non-shanty residents with residential moves**

(A) Sequence index plot for levels of access to water of shanty residents: individual housing trajectories as horizontal lines grouped by initial conditions. (B) Parallel-coordinate plot for levels of access to water for shanty residents: line thickness shows volume of flow for a given housing trajectory. (C) Sequence index plot for levels of access to water of non-shanty residents: individual housing trajectories as horizontal lines grouped by initial conditions. (D) Parallel-coordinate plot for levels of access to water for non-shanty residents: line thickness shows volume of flow for a given housing trajectory.

**
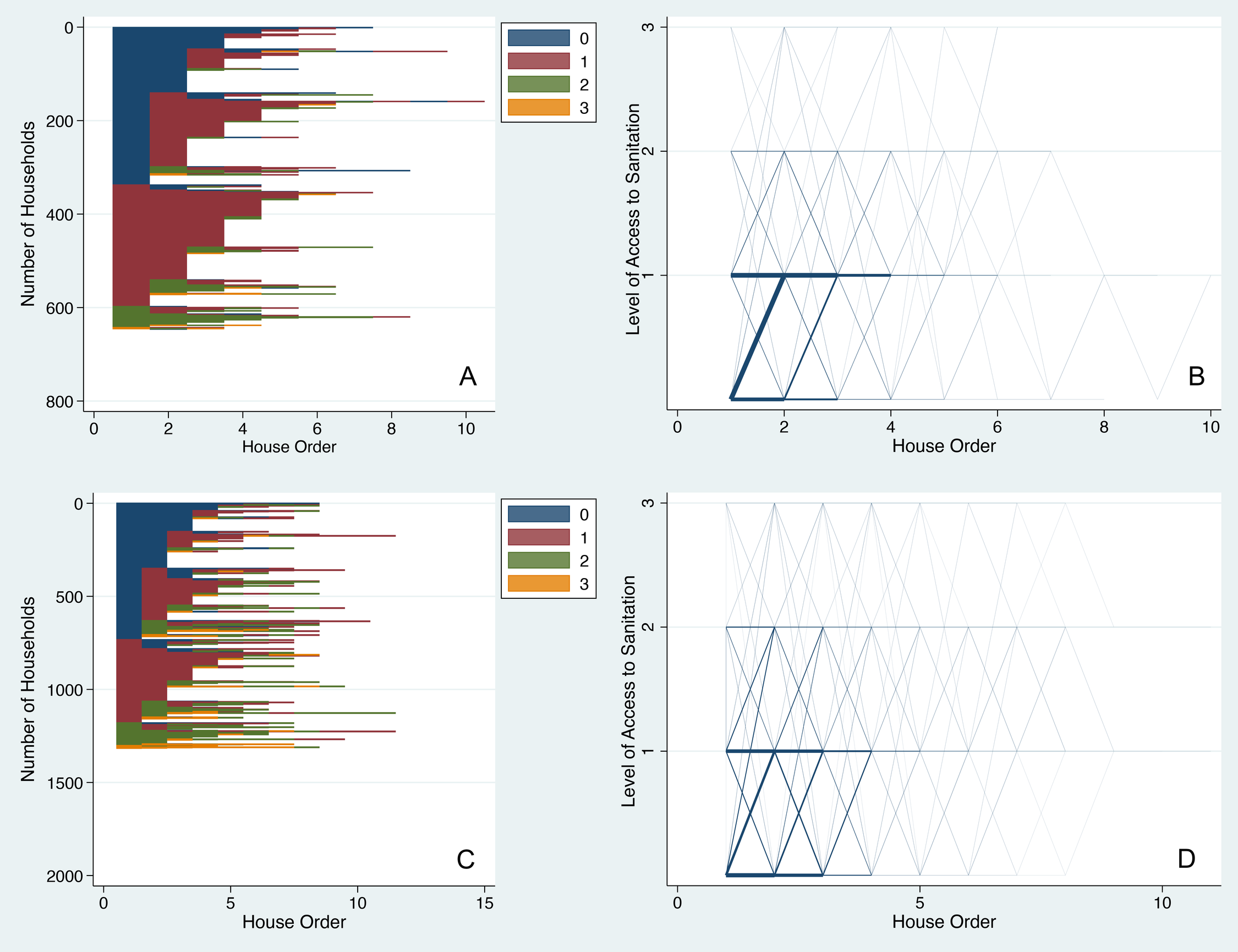
**

**Fig S2.2. Dynamics of access to sanitation for shanty and non-shanty residents with residential moves**

(A) Sequence index plot for levels of access to sanitation of shanty residents: individual housing trajectories as horizontal lines grouped by initial conditions. (B) Parallel-coordinate plot for levels of access to sanitation for shanty residents: line thickness shows volume of flow for a given housing trajectory. (C) Sequence index plot for levels of access to sanitation of non-shanty residents: individual housing trajectories as horizontal lines grouped by initial conditions. (D) Parallel-coordinate plot for levels of access to sanitation for non-shanty residents: line thickness shows volume of flow for a given housing trajectory.

**
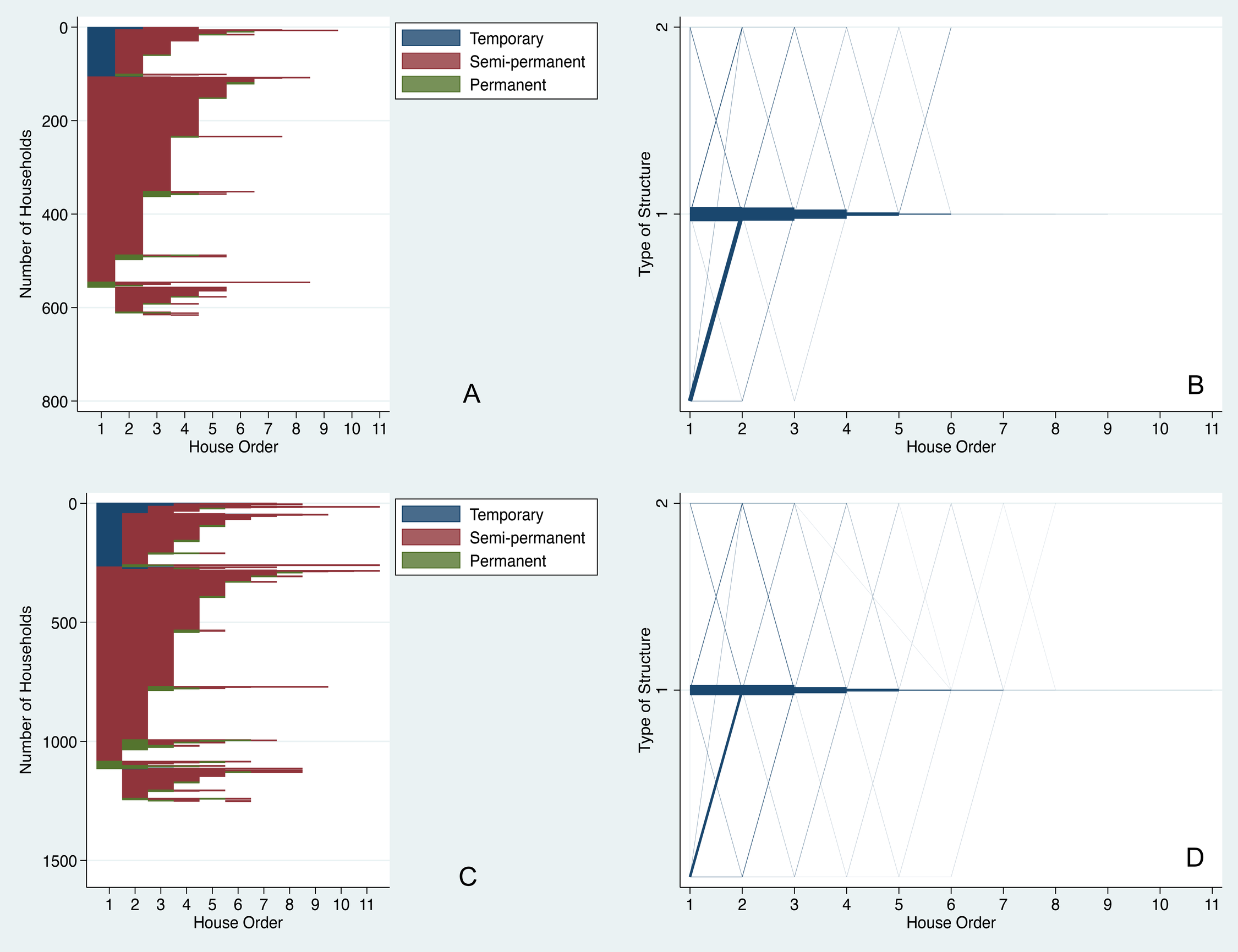
**

**Fig S2.3. Dynamics of housing with durable structure for shanty and non-shanty residents with residential moves**

(A) Sequence index plot for levels of durable structure for shanty residents: individual housing trajectories as horizontal lines grouped by initial conditions. (B) Parallel-coordinate plot of durable structure for shanty residents: line thickness shows volume of flow for a given housing trajectory. (C) Sequence index plot for durable structure for non-shanty residents: individual housing trajectories as horizontal lines grouped by initial conditions. (D) Parallel-coordinate plot for durable structure for non-shanty residents: line thickness shows volume of flow for a given housing trajectory.

**
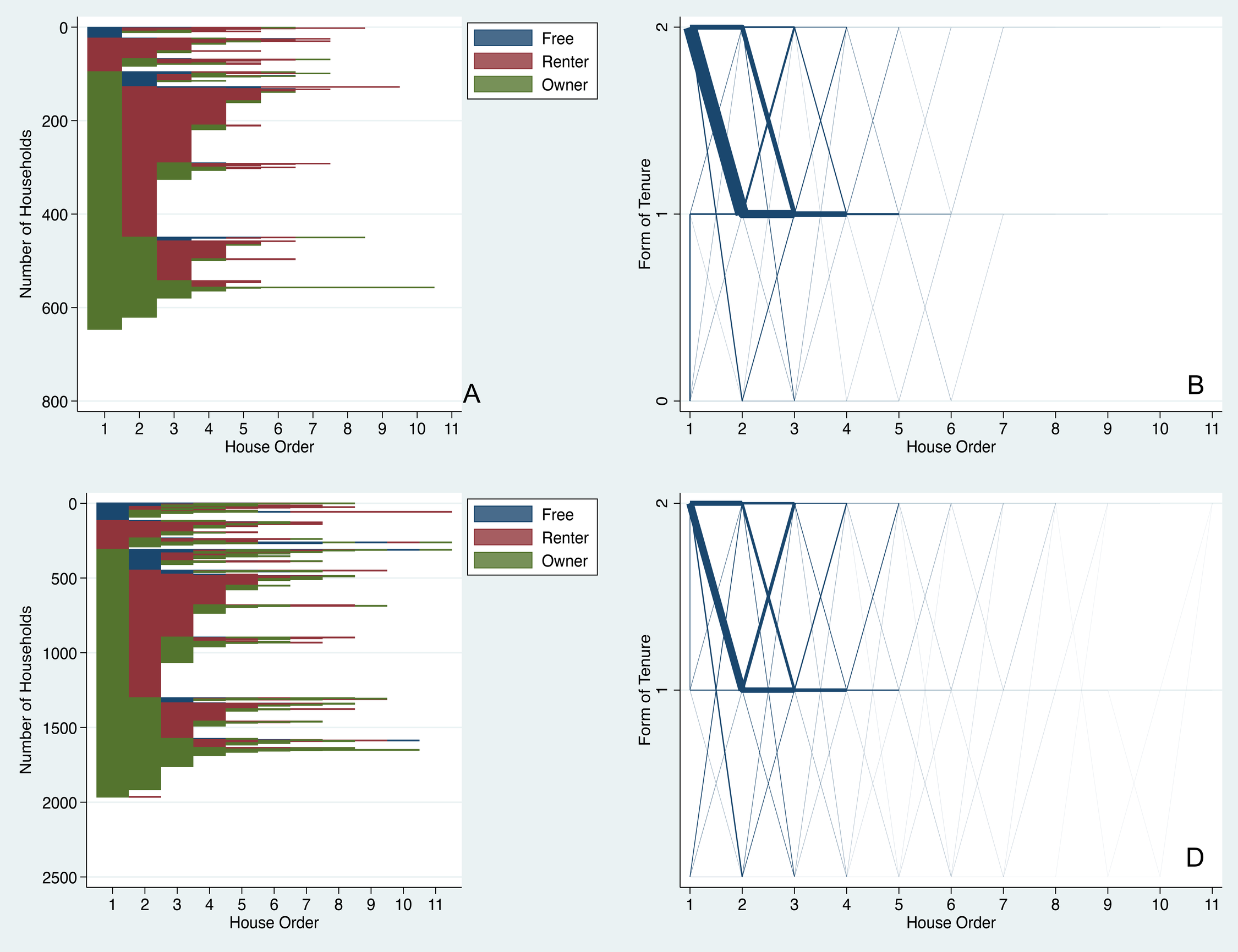
**

**Fig S2.4. Dynamics of tenure forms for shanty and non-shanty residents with residential moves**

(A) Sequence index plot for forms of tenure for shanty residents: individual housing trajectories as horizontal lines grouped by initial conditions. (B) Parallel-coordinate plot of forms of tenure for shanty residents: line thickness shows volume of flow for a given housing trajectory. (C) Sequence index plot of forms of tenure for non-shanty residents: individual housing trajectories as horizontal lines grouped by initial conditions. (D) Parallel-coordinate plot of forms of tenure for non-shanty residents: line thickness shows volume of flow for a given housing trajectory.
