# Supplementary material for: Effects of residential mobility and migration on standards of living in Dar es Salaam, Tanzania: A life-course approach
Source: PLoS One. 2020 Sep 29;15(9):e0239735. doi: 10.1371/journal.pone.0239735 (PMC7523954; doi:10.1371/journal.pone.0239735)
Supplement: Attachment — Submitted filename: Response to Reviewers.docx [file pone.0239735.s006.docx]

Response to Reviewers

Reviewers’ Responses to Questions

1. If the authors have adequately addressed your comments raised in a previous round of review and you feel that this manuscript is now acceptable for publication, you may indicate that here to bypass the “Comments to the Author” section, enter your conflict of interest statement in the “Confidential to Editor” section, and submit your "Accept" recommendation.

Reviewer #1: (No Response)

Reviewer #2: All comments have been addressed

**Authors’ Response:** Thank you.

2. Is the manuscript technically sound, and do the data support the conclusions?

Reviewer #1: Yes

Reviewer #2: Yes

**Authors’ Response:** Thank you.

3. Has the statistical analysis been performed appropriately and rigorously?

Reviewer #1: Yes

Reviewer #2: Yes

**Authors’ Response:** Thank you.

4. Have the authors made all data underlying the findings in their manuscript fully available?

Reviewer #1: Yes

Reviewer #2: Yes

**Authors’ Response:** Thank you.

5. Is the manuscript presented in an intelligible fashion and written in standard English?

Reviewer #1: Yes

Reviewer #2: Yes

7. PLOS authors have the option to publish the peer review history of their article ([what does this mean?](https://journals.plos.org/plosone/s/editorial-and-peer-review-process#loc-peer-review-history)). If published, this will include your full peer review and any attached files. If you choose “no”, your identity will remain anonymous, but your review may still be made public. Do you want your identity to be public for this peer review? For information about this choice, including consent withdrawal, please see our [Privacy Policy](https://www.plos.org/privacy-policy).

Reviewer #1: **Yes:** Rory Coulter

Reviewer #2: No

**Authors’ Response:** Thank you for your time and very careful review of both the versions of this manuscript. We cannot thank you enough for your constructive feedback.

Response to Reviewers’ Detailed Comments:

**Comments from Reviewer 1**

**Comment 1:** This is my second reading of this manuscript and it is pleasing to see that the authors have attended to the issues raised in my previous review. A better case is built for doing a longitudinal analysis and the discussion of life course perspectives is much improved. I also appreciate the use of a priori sequence ‘types’ that give much greater clarity to the analysis. Taken together, these changes have produced a much-improved paper. Good work.

**Response:** Thank you very much for carefully reviewing our manuscript and providing very constructive feedback – twice. We really appreciate it.

**Comment 2:** There are a few small changes I’d suggest the authors consider in their revision:

P3 - can you give a local example or two of the types of policies mentioned here?

**Response:** Thank you very much for this wonderful suggestion. We have added a new discussion to substantiate our point on temporal dimensions and provided a reference that has multiple examples of spatially oriented slum policies.

**Comment 3:** P5 – it would be worth mentioning the gendered nature of migration here. Developed world research has identified women as ‘tied movers/stayers’ (Cooke 2008 in Population, Space and Place) and the issue of ‘left-behind’ families is a key one across much of the Global South. The basic point to note here is that the positive wage benefits of migrating may accrue only to certain household members.

**Response:** We are grateful for this constructive suggestion. Thank you for sharing the resources on this subject. We have now added these points in our discussion.

**Comment 4:** P6 – Household ageing is a problematic idea when households are made up of varying persons. Try and make sure the paper consistently notes that your analysis is of individual life course and housing careers.

**Response:** Thank you very much for pointing this out. We have thoroughly reviewed our manuscript to make sure that we consistently use individuals/people as opposed to households and household members.

**Comment 5:** P9 – RQ2 implies causality and that cannot be tested here. Avoid causal language unless justified by your research approach (e.g. the use of experimental rather than observational data).

**Response:** Thank you very much for providing this wonderful suggestion. We agree that our research approach does not warrant a discussion on causality. We have revised the second research question to ensure that it is in congruence with our analysis.

**Comment 6:** Sequence plots – I still find these rather messy and unhelpful. For example, I struggle to see from them what the most common patterns of sequence are (as described in the text). Can they be improved in some way?

**Response:** Thank you very much for your suggestion. We believe that part of the problem is that our data is not a balanced panel (with varying numbers of housing careers resulting in varying lengths of history), unlike a typical use case for sequence index plots. Second, the graphs present a rather large sample (many textbook examples we checked have fewer individuals). We have used parallel coordinate plots in conjunction with sequence index plots to aid with visualization of the most common patterns. We also added a description in the captions of these figures to clarify what each of these panels represent in our earlier revision. We agree that figures have limited utility, but tables provide the frequency of the most common patterns with numeric precision to overcome that limitation.

**Comment 7:** Sequence plots – the n here looks to be <2000 for most of the figures. This makes sense when subgroup analyses are conducted but not when whole sample descriptives are presented (e.g. Figures 1-2). Why is the n < 2397?

**Response:** Thank you for pointing this out. Sequences with gaps in history in a variable of interest (e.g., water) are excluded from the analysis. We have clearly identified this limitation in the data section and reported missing values in residential history. These gaps in history explain the lower N in sequence plots.

**Comment 8:** Migrant and shanty sequence analysis – rather than presenting so many plots, I wonder whether crosstabs of migrant/shanty status (x) against sequence type (y) would be easier to read? They would also enable statistical comparisons to be drawn – e.g. to see whether more migrants than stayers are ‘perpetually deprived’.

**Response:** Thank you very much for this excellent point. We have added the suggested tables that enable statistical comparison. We have revised the text to refer to the content of the table. We have moved Figures 5 to 12 to the supplemental materials section and referred them in our revised text.

**Comment 9:** Figure 14 is rather hard to read.

**Response:** Thank you very much for pointing this out. We have increased the font size of graphs and improved the layout. We believe that the system-generated PDF does not include high-resolution images. We have made sure that the image is readable in its final form.

**Comments from Reviewer 2**

**Comment 1:** The authors have addressed my comments comprehensively and within the limitations of the data. The authors have undertaken significant revisions which have greatly improved the paper. Generally, I think this manuscript is ready for publication.

**Response:** Thank you very much for your kind words and very constructive feedback on our manuscript.

**Comment 2:** I have a couple of small comments:
l. 221: Dar es Salaam is not home to 40% of Tanzania's population. The total population of Tanzania was 44.9 million according to the 2012 census, whereas in Dar es Salaam region it was 4.4 million

**Response:** Thank you very much for pointing out this error. We missed writing Tanzania’s urban population. We have corrected it now and cited the source of this information.

**Comment 3:** l. 264: Why the date and time stamp in the text?

**Response:** Thank you very much for pointing this out. Our reference management tool, Zotero, sometimes erroneously replaces cited references with a time stamp. In this version, we have removed all automatically updating fields to make sure that our final version doesn’t have any such typographical errors.
